# Commemorating Misadventures, Celebrating Collaborations

**DOI:** 10.3201/eid2402.AC2402

**Published:** 2018-02

**Authors:** Byron Breedlove

**Affiliations:** Centers for Disease Control and Prevention, Atlanta, Georgia, USA

**Keywords:** art science connection, emerging infectious diseases, art and medicine, about the cover, Pablo Picasso, Don Quijote, Don Quixote, commemorating misadventures, celebrating collaborations, horses, equids, viruses, transmission, anthrax, cryptosporidiosis, Eastern equine encephalitis, Western equine encephalitis, Venezuelan equine encephalitis, glanders, Hendra virus disease, leptospirosis, rabies, zoonoses

**Figure:**
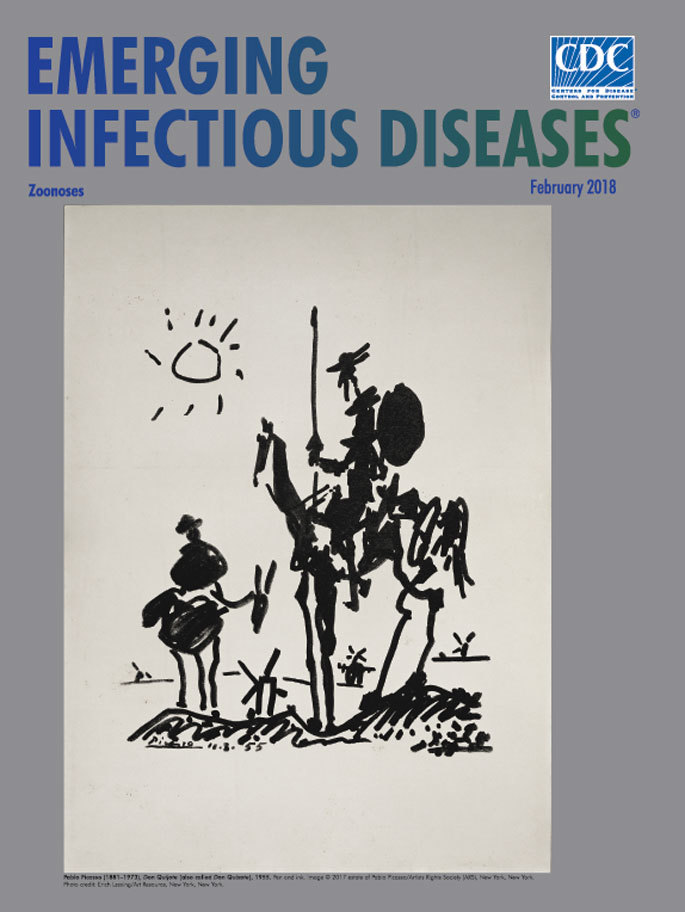
**Pablo Picasso (1881–1973). *Don Quijote* (also called *Don Quixote*), 1955.** Pen and ink image. © 2017 Estate of Pablo Picasso/Artists Rights Society (ARS), New York, New York, USA. Photo Credit Erich Lessing/Art Resource, New York, New York

“What is more dangerous than to become a poet? which is, as some say, an incurable and infectious disease.”

―Miguel de Cervantes Saavedra, Don Quixote

For the first half of the 20th century, Spanish artist Pablo Picasso dominated the art world in a great variety of creative styles and forms, creating more than 20,000 paintings, prints, drawings, sculptures, and other works. His widely recognized and now iconic black and white sketch of Don Quixote proved a departure from his intricate surreal and cubist paintings. This briskly rendered drawing first appeared on the cover of the August 18–24, 1955, issue of the journal *Les Lettres Françaises.* That issue commemorated the 350th anniversary of the publication of *Don Quixote*, Part I, by Miguel de Cervantes Saavedra in 1605. Together with Part II (1615), this publication is considered among the greatest, most influential works of Western fiction.

In this drawing, Picasso conveys the pervasive lassitude and weariness that readers associate with Cervantes’s characters and their series of hopeless quests. The essence of the pair’s relationship is distilled in this simple, abstract sketch. Don Quixote de la Mancha dominates the drawing. Picasso portrays him sitting astride his horse, Rocinante, gazing straight ahead. Sancho Panza, Don Quixote’s loyal squire, sits much lower on his donkey, Dapple, and tilts his head up toward Don Quixote. The idealistic Don Quixote scans the windmills dotting the horizon, pondering his next adventure while the faithful and practical Sancho Panza, who strives to reason with him, has resigned himself to yet another hapless foray. A lopsided sun floats in the upper left of the drawing. Several windmills stand low on the horizon of the La Mancha plateau, and another juts between the two figures.

Cervantes scholar Anthony George Lo Ré characterizes these figures as “almost laconic and deformed.” He elaborates: “The knight’s head, capped by what would be Mambrino’s helmet, is connected to his shoulders by a neck made with a single, thin line, and it sports a pointed nose and a long, equally thin goatee. He carries a lance in his right hand and the reins and a circular shield apparently in his left. Rocinante is the bag of bones described by Cervantes: long, thin forelegs, a haunch that looks transparent for the right thigh can be seen behind the left, and with rough lines and shading that suggest girth, loin, croup, and saddle. Sancho appears to the left, a black mass vaguely defining his round body, and sitting on Dapple who has a long, wiry neck and thin, long ears.”

Rocinante and Dapple appear in all of the novel’s various quests, and Picasso portrays man and steed as being inextricably connected. Writer Jon Katz notes that “Without Rocinante and Dapple, *Don Quixote* is hardly a book at all. . . .The two equine companions are mirrors of the men who ride them into every imaginable predicament and misadventure.” That human–equine relationship, integral to both the novel and drawing, fits within an extensive, diverse, and evolving history long chronicled in prehistoric cave art, myths, literature, song, and film.

Recently discovered archeological evidence places the earliest domestication of horses as beginning somewhere in the mid–fourth millennium bce. Though horses may have initially been a source of food, their domestication profoundly advanced human transportation, agriculture, communication, and warfare. In much of the world today, leisure and competitive riding, often at large-scale events, has supplanted earlier uses, and contemporary human interactions with horses frequently involve riders, caretakers, veterinarians, and therapists.

A wide range of infectious diseases affect horses, caused by organisms varying from common equine herpesviruses to a newly identified parvovirus associated with serum hepatitis reported in this issue. Some of those diseases can cause significant economic losses to local economies as well as the global horse industry. Another foreseeable consequence from those many close connections between horses and humans is the potential for myriad zoonotic conditions, including anthrax, cryptosporidiosis, Eastern equine encephalitis, Western equine encephalitis, and Venezuelan equine encephalitis, glanders, Hendra virus disease, leptospirosis, and rabies. 

Misadventurers Don Quixote and Sancho Panza were blissfully unconcerned about matters such as zoonotic infections when they trotted off on their humble mounts toward the next windmill on the horizon. Their solitary saga, frequently cited for its futility, contrasts with collaborative approaches such as the One Health Initiative, which emphasizes interdisciplinary collaboration for protecting and preserving human and animal health.
